# Early Dynamics of Hepatitis B Virus (HBV)-DNA and Surface Antigen (HBsAg) in Ramp-Up Phase of Viremia: Implications for Performance Evaluation of Blood Screening Assays

**DOI:** 10.3390/v14091942

**Published:** 2022-08-31

**Authors:** Harry van Drimmelen, Nico Lelie

**Affiliations:** 1 Biologicals Quality Control (BioQControl), De Droogmakerij 31h, 1851LX Heiloo, The Netherlands; 2 Lelie Research, Parkstraat 2, 1811DK Alkmaar, The Netherlands

**Keywords:** HBV-DNA, HBsAg, seroconversion panel, standard dilutions, analytical sensitivity, genotype

## Abstract

The Common Specifications/EU 2017/746 regulation for market approval of class D in vitro diagnostic devices (IVDs) intended for detection of blood borne viruses requires testing of the International Standard and 10–30 seroconversion panels to demonstrate ‘state of the art’ assay performance. We examined whether these requirements for performance evaluation are reasonable for HBV-DNA and HBsAg assays. For this purpose, we quantified HBsAg and HBV-DNA (genotype A) in the ramp-up phase of five seroconversion panels and demonstrated a remarkably parallel increase in the Log concentration of both analytes over time. Testing of seroconversion panels by three nucleic acid amplification technology (NAT) methods in multiple replicates and probit analysis with sufficient critical samples from all five panels taken together showed detection limits in copies/mL that were comparable to those on a HBV-DNA genotype A standard dilution panel. This indicates that the viral doubling time in the ramp-up phase is equal above and below the quantification limit of the viral load assay. The geometric mean HBsAg (PRISM) cutoff crossing point was 20 days later than the 50% NAT (Ultrio Plus) conversion point equivalent to 1500 (range: 1100–2200) and 4.8 (CI: 3.7–6.4) HBV-DNA copies/mL, respectively. Analytical sensitivity data of different NAT assay versions obtained over a decade demonstrated that the detection limit on the International Standard is not representative of all genotyped reference samples. From our detailed mathematical analysis, we conclude that HBV-DNA and HBsAg standard dilution series are functionally equivalent to seroconversion panels. A general requirement of a 95% detection limit ≤100 HBV-DNA copies/mL for different viral genotypes would be a better-defined regulation for EU market approval of NAT blood screening assays than the testing of multiple seroconversion panels to claim ‘state of the art’ performance.

## 1. Introduction

At the time of the introduction of hepatitis B virus (HBV)-DNA screening of blood donations, both seroconversion panels and analytical sensitivity panels (standard dilution series) were used in a performance evaluation study of the first automated nucleic acid amplification technology (NAT) screening systems, i.e., the Ultrio assay on the Tigris instrument and the TaqScreen 1.0 assay on the cobas S201 platform [1,2]. After the performance evaluation data of these first-generation multiplex NAT systems had been reported by Assal and coworkers [1,2], additional testing of the same HBV seroconversion and HBV standard dilution panels had been performed in multiple replicates by the Ultrio and the more sensitive Ultrio Plus assay version by Dr. J. Linnen and coworkers (Gen-Probe, currently Grifols Diagnostic Solutions Inc., San Diego, CA, USA). Because the quantitative HBV-DNA results in both the seroconversion and analytical sensitivity panels had been calibrated against the same reference standard in copies/mL, we were able to compare the assay seroconversion points with the limits of detection (LOD) in the standard dilutions. For the seroconversion panels, this was mathematically possible by extrapolating HBV-DNA concentrations in the early ramp-up phase from the observed exponential increase of viral load in later sequential samples. The HBsAg-positive samples in the seroconversion panels were tested quantitatively by two HBsAg assays (Abbott PRISM and BIO-RAD Monolisa Ultra) against a calibration curve of the second WHO HBsAg (00/588) standard. This enabled us to compare the course of the HBV-DNA and HBsAg growth curves during the ramp-up phase of viremia as well as to calculate the ratios between potentially infectious HBV virions (so-called Dane particles) and subviral (noninfectious) 20 nm HBsAg particles.

In addition to comparing the reactivity rates of the different HBV NAT methods on HBV standard dilution and seroconversion panels of the same genotype (A2), we compared the 95% and 50% LODs on dilution panels of different cross-calibrated HBV genotype standards which had been tested in the same experiments. In the following decade, we also compared the LODs of newer NAT assay versions on the same HBV genotype standards [3,4] as well as on dilution panels prepared from the Eurohep standard [5], the 2nd WHO 97/750 genotype A standard [3,4], and members of the WHO HBV genotype reference panel [6].

The results in this manuscript are used to discuss the value of seroconversion panels and analytical sensitivity panels of different genotypes for performance evaluation of blood screening assays. In the context of our findings, we discuss the criteria for performance evaluation of blood screening assays in the recently adopted Common Specifications (CS) [7] which now becomes a legal requirement for market approval of in vitro diagnostic devices (IVDs) with the transition to the new European Union (EU) IVD Regulation 2017/746 (IVDR) [8].

## 2. Materials and Methods

### 2.1. Assays on HBV Seroconversion Panels

In a head-to-head comparison study of the Etablissement Français du Sang (EFS, France)^2^, HBV seroconversion panels #6284, #6289, #6292, #11006, and #11008 (Zeptometrix, Buffalo, NY, USA) had been tested in 4 replicates by the old Ultrio assay (Grifols Diagnostic Solutions, San Diego, CA, USA) and TaqScreen 1.0 assay (Roche Molecular Systems, Pleasanton, CA, USA), the latter in 1:6 dilution to mimic minipool of 6 (MP6) testing. At that time, the panels were also tested in the Bayer Versant bDNA 3.0 assays by Dr. M. Koppelman (Sanquin Diagnostic Services, Amsterdam, The Netherlands) in parallel with dilutions of the S0011 VQC-Sanquin HBV genotype A2 standard containing 2.15 × 10^9^ copies/mL (as historically quantified using the bDNA 3.0 assay) [9]. The geometric mean conversion factor of VQC copy to measured bDNA 3.0 copy in replicate tests was 1.06 (range: from 1.02 to 1.12). As the measured values were close to the nominal reference values of the S0011 HBV genotype A2 standard used for preparing the PeliCheck dilution panel (see below), it was decided not to adjust the quantitative bDNA 3.0 results in the seroconversion panel members. Viremic samples in the five HBV seroconversion panels were sequenced in the S region by Dr. Koppelman (Sanquin Diagnostic Services, Amsterdam, The Netherlands) and all seroconverting plasma donors were found to be infected with HBV genotype A2.

The members of the five seroconversion panels were also tested by EFS in the Abbott PRISM HBsAg assay in triplicate and BIO-RAD Monolisa HBsAg Ultra assay in duplicate in parallel with a dilution series of the 2nd WHO HBsAg standard (00/588), tested in duplicate on two days [2]. The WHO HBsAg standard calibration curve allowed for transformation of the HBsAg S/CO response values into International Units (IU/mL) and nanogram (ng)/mL using a conversion factor of 0.67 ng/IU [10,11,12].

At the time of the introduction of the more sensitive Ultrio Plus assay, the manufacturer (Gen-Probe, San Diego, CA, USA) used the same five HBV seroconversion panels for comparing the sensitivity with the previous Ultrio assay version. For this purpose, some of the seroconversion panels were tested in as many as 16–18 replicate Ultrio and Ultrio Plus tests while others had been tested in only 4 replicates. The availability of a high amount of replicate test results on seroconversion samples with very low viral load allowed for a mathematical analysis in which the NAT seroconversion points could be compared with the LOD on the HBV genotype A2 standard dilution series (see below).

### 2.2. Assays on Analytical Sensitivity Panels of Different HBV Genotypes

Ten-member PeliCheck standard dilution panels of different HBV genotypes had been developed originally by VQC-Sanquin (currently Biologicals Quality Control (BioQControl), Heiloo, The Netherlands) and were calibrated in genome equivalents (geq)/mL based on the Chiron branched DNA (bDNA) 1.0 assay. Each panel was composed of 10 members containing 10,000, 3000, 1000, 300, 100, 30, 10, 3, 1, and 0.3 geq/mL, respectively. The PeliCheck HBV reference panels S2384 (genotype A), S2385 (genotype B), S2386 (genotype C), S2387 (genotype D), S2388 (genotype E), S2389 (genotype F), and S2390 (genotype G) were tested in 12-replicate Ultrio and TaqScreen 1.0 assays in the previously published head-to-head comparison study of EFS [1]. The same batches of reference panels were later used for performing an additional 12-replicate tests in both Ultrio and the more sensitive Ultrio Plus assay by Dr. J. Linnen (Gen-Probe, currently Grifols Diagnostic Solutions, San Diego, CA, USA). The replicate test results on the PeliCheck S2384 HBV genotype A2 panel could be compared with those on the 5 seroconversion panels because the tests on these panels were performed during the same series of experiments by EFS and Gen-Probe laboratories.

### 2.3. Calibration of HBV Genotype Standard Dilution Panels in Copies/mL and IU/mL

The PeliCheck S2384 HBV genotype A panel was prepared from the S0011 VQC-Sanquin genotype A2 standard containing 3 × 10^9^ geq/mL based on original quantification in the bDNA 1.0 assay. We later recalibrated this standard to 2.15 (2.11–2.20) 10^9^ copies/mL using 28 tests performed over time in a later (3rd) version of the bDNA assay (Bayer Versant bDNA 3.0) [9]. This value was comparable to 2.11 (2.05–2.17) 10^9^ copies/mL as quantified by 198 Amplicor Monitor tests performed during the same period [9]. Dilutions of the S0011 VQC-Sanquin HBV genotype A standard were also calibrated against the first WHO HBV 97/746 standard [13] using 12–16 replicate tests per standard dilution by Dr. T. Cuypers (Sanquin Diagnostic Services, Amsterdam, The Netherlands), which resulted in a conversion factor (95% confidence interval (CI)) of 5.33 (5.11–5.55) copies/IU.

The HBV standards of genotype B, C, D, E, F, and G in the PeliCheck panels were also recalibrated against the S0011 VQC-Sanquin HBV genotype A standard in three different bDNA 3.0 runs, each in triplicate tests (total 9 tests per standard) [9]. The adjusted concentrations in the PeliCheck analytical sensitivity panel members in copies/mL were then used to determine the 50% and 95% LOD by probit analysis.

### 2.4. Testing of Additional HBV-DNA Genotype Standard Dilution Panels

In the following decade (2007–2018), we prepared new lots of HBV genotype standard dilution panels from the same standards as used for the PeliCheck analytical sensitivity panels mentioned above. In addition, dilution panels were prepared from different international standards, i.e., the Eurohep genotype A and D standard [5], the 97/750 genotype A standard [13], and the members of the WHO HBV-genotype reference panel [6]. We independently cross-calibrated these standards in multiple bDNA 3.0 tests and found results similar to those reported by Chudy et al. [6,9]. The replicate NAT results obtained by several laboratories which tested the HBV genotype standard dilution panels were collected until 2018, and the 95% and 50% LODs with different Ultrio and cobas MPX assay versions were calculated by probit analysis using statistical analysis software package SPSS version 17.0.

### 2.5. Mathematical Analyses on Seroconversion and Dilution Panel Data

#### 2.5.1. Regression Analysis on HBV-DNA and HBsAg Concentration in Ramp-Up Phase

Measured HBV-DNA concentrations in the ramp-up phase of viremia were used to back estimate the viral load in earlier seroconversion samples below the quantification limit of the bDNA 3.0 assay. The method was based on a log-linear ramp-up phase model [14,15,16,17] where the log viral load is plotted against time. The formula is: ln (copies/mL) = a.X + b (where X is the number of follow up days and a and b are constants). The regression lines were based on 4–6 ramp-up phase samples per panel with increasing HBV-DNA concentrations in the bDNA 3.0 assay. The doubling time could be calculated from the regression line with the following formula:

Doubling time = days interval/^2^log (conc_end_/conc_start_)

For each of the HBV panels, their own estimated viral doubling time was used to back estimate the viral load in the previous bleeds and, thus, arbitrarily determine the start of the potentially infectious window period at a viral concentration of 1 copy/20 mL plasma (t = 0).

For the quantification of HBsAg against the WHO standard, a Logit-Log regression model method was used. The Logit transformation of the HBsAg signal (S or S/CO value) is:

ln((S_max_ − S)/(S − S_min_))

where S_max_ is the signal at the saturation point of the HBsAg assay and S_min_ is the signal of the negative population. Both S_min_ and S_max_ are estimated by iteration to the best fit. The effect of the Logit transformation is that a linear relation is obtained between Logit (S/CO) and Log HBsAg concentration in IU/mL. The concentration at the cutoff crossing point in the WHO HBsAg standard dilution series was then used to estimate the time point where the HBsAg signal crosses the cutoff in the seroconversion panels. For this, we used the linear relationship between time and Log IU/mL HBsAg in the ramp-up phase samples starting with the samples at (just above or below) cutoff level and ending near saturation of signal and peak viremia (excluding samples with HBV-DNA concentration ≥2 × 10^7^ copies/mL in our study). The time estimate at the HBsAg cutoff crossing point was then used to calculate the HBV-DNA concentration.

#### 2.5.2. Correlation between HBV-DNA and HBsAg Concentration in Ramp-Up Phase

By comparing the Log HBV-DNA and Log HBsAg concentration in the ramp-up phase samples, we were able to determine the correlation coefficient by linear regression analysis, as well as the ratio between the concentration of HBV-DNA in copies/mL and HBsAg in IU/mL (or HBV-DNA copies per IU of HBsAg). Historically, we established that one IU of the 2nd WHO standard 00/588 (which was used for quantification of HBsAg in the ramp-up phase samples) corresponded with 0.67 nanogram or Paul Ehrlich standard (PEI) units, according to calibration using the old Abbott Ausria assay [10]. According to old experiments performed by Prof W. Gerlich, one nanogram or PEI unit corresponds to approximately 2 × 10^8^ HBsAg particles [18,19]. As one HBV-DNA copy corresponds to one potentially infectious virus, the ratio between the number of virions and subviral particles could be calculated for all ramp-up phase samples in the 5 panels taken together.

#### 2.5.3. Estimation of HBV-NAT and HBsAg Seroconversion Point in Ramp-Up Phase

For each of the seroconversion panel members, the HBV-DNA concentration below the quantification limit of the bDNA 3.0 assay was extrapolated from the Log-linear regression line. The proportion of reactive replicate tests in Ultrio, Ultrio Plus, and TaqScreen 1.0 (in 1:6 diluted tested seroconversion samples) was plotted against the calculated Log HBV-DNA concentration. Then, the data of the five seroconversion panels were combined to have sufficient critical samples for establishing the probit curves of the three NAT methods on the Log HBV-DNA concentrations in the ramp-up phase samples. The 50% LOD and conversion time point for each of the NAT methods was determined separately and compared with the geometric mean viral load at the HBsAg cutoff crossing time points in the five panels.

#### 2.5.4. Comparison of 50% LOD on Seroconversion and Standard Dilution Panel

The 50% LOD (NAT conversion point) for each of the assays in the combined dataset of the five seroconversion panels were compared with the 50% LOD (95% CI) on the S2384 PeliCheck HBV-DNA genotype A standard dilution series by parallel line probit analysis using SPSS 17.0 software. This was possible because the two types of panels were tested in the same set of experiments by the laboratories and were calibrated against the same reference standard. If the 50% LODs on the two types of panels are comparable, it is an indication that the Log-linear ramp-up phase model is valid and that the HBV-DNA doubling time is the same in the very early ramp-up phase before HBV-DNA becomes quantifiable by the bDNA3.0 assay.

#### 2.5.5. Analytical Sensitivity Analysis of NAT Assays on HBV Genotype Panels

The 50% and 95% LOD in the PeliCheck HBV-DNA standard dilution panels for genotype A, B, C, D, E, F, and G were calculated by probit analysis using the SPSS 17.0 statistical package. For this probit analysis, the data sets of the six genotypes were combined in a parallel line model. However, to calculate the relative sensitivity factor (and 95% CI) of Ultrio Plus against TaqScreen 1.0, the data from the two methods on one genotype panel were used for probit analysis in a parallel line model. The 95% and 50% LODs on later-manufactured HBV genotype panels by BioQControl were determined individually for the Ultrio, Ultrio Plus, Ultrio Elite, TaqScreen 1.0, TaqScreen 2.0, and cobas MPX assays using the cumulative data collected over time.

## 3. Results

### 3.1. Course of HBV-DNA and HBsAg Concentration in Ramp-Up Phase

Linear regression analysis on Log HBV-DNA concentration versus time in the five seroconversion panels (measured on 4–6 viremic ramp-up phase samples with quantifiable viral load in the bDNA 3.0 assay per panel) showed doubling times of 1.81 to 2.75 days (mean 2.44 days) (Table 1). Assuming that the same doubling time and Log-linear increase of HBV-DNA were also present at concentrations below the bDNA 3.0 quantification limit, we calculated the time point when 1 HBV-DNA copy would be present in 20 mL plasma, i.e., the assumed plasma volume in a Red Blood Cell (RBC) transfusion. This time point in the seroconversion panel series was set as the start of the infectious window period (day 0) and used for calculation of HBV-DNA and HBsAg conversion time points (Table 1).

**Figure 1:**
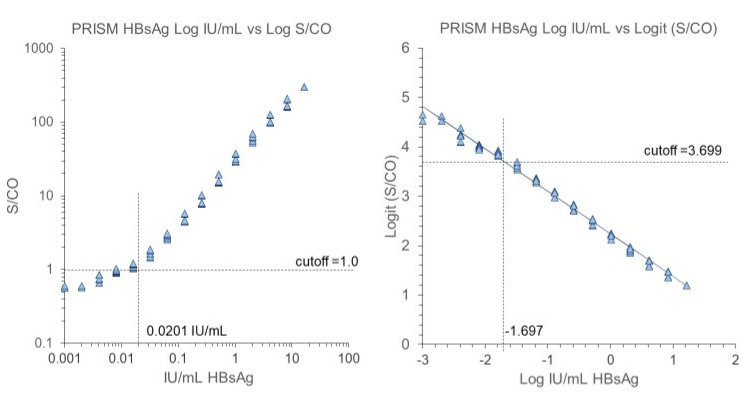
Calibration curves of twofold WHO 00/588 HBsAg standard dilutions tested twice in duplicate in HBsAg PRISM assay showing better linearization of the regression line until cutoff level when plotting Log IU/mL against Logit S/CO ratio (**right** graph) than Log IU/mL against Log S/CO (**left** graph).

**Figure 2:**
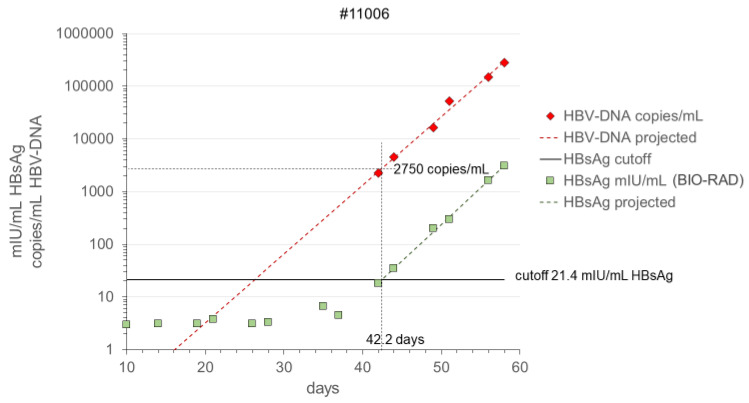
Example of course of Log HBV-DNA concentration (copies/mL in bDNA 3.0 assay) and Log HBsAg concentration (mIU/mL in BIO-RAD assay) in ramp-up phase of viremia (seroconversion panel #11006).

**Figure 3:**
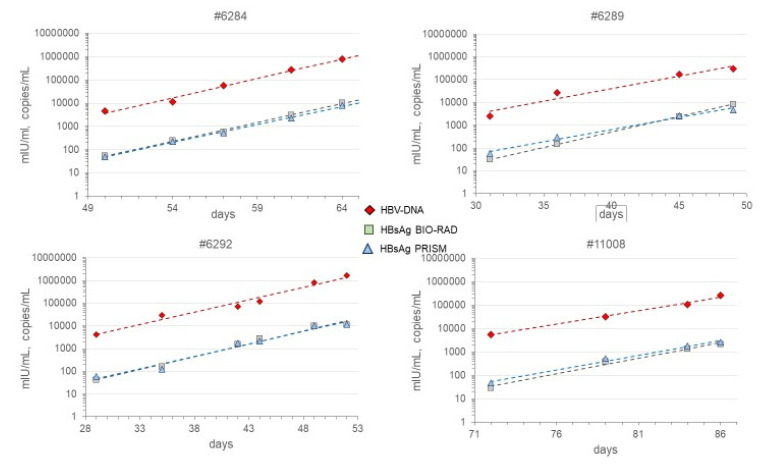
Parallel increase of Log HBV-DNA (bDNA 3.0 assay) and Log HBsAg concentration (PRISM and BIO-RAD assays) during ramp-up phase of viremia in seroconverting plasma donors (seroconversion panels #6284, #6289, #6292, and #11008).

**Figure 4:**
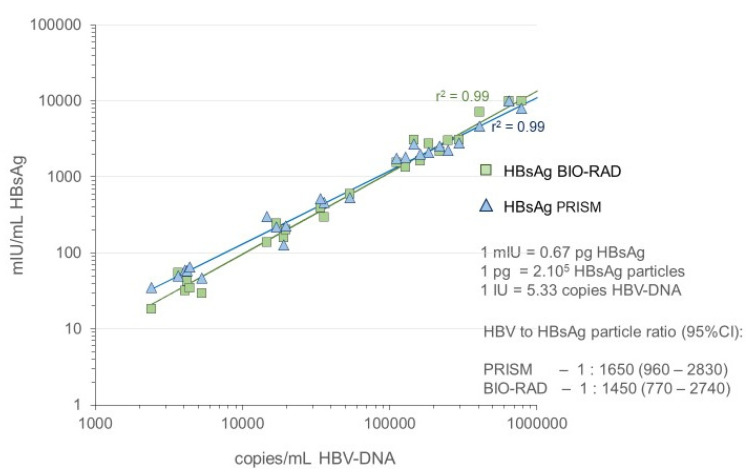
Correlation between Log copies/mL HBV-DNA (bDNA 3.0 assay) and Log mIU/mL HBsAg (PRISM and BIO-RAD assays) measured with 25 ramp-up phase samples from five seroconversion panels (#6284, #6289, #6292, #11006, and #11008) and estimation of ratio between number of HBV virions (HBV-DNA copies) and subviral 20 nm HBsAg particles.

The HBsAg S/CO values on the seroconversion samples in the PRISM and BIO-RAD Monolisa HBsAg assays were compared with calibration curves of the WHO HBsAg 00/588 genotype A standard for which a dilution series was tested twice in duplicate in both assays. Figure 1 shows that the best fit for a linear regression line to cutoff level was obtained by plotting Logit S/CO versus Log IU/mL HBsAg; this fit was better than plotting Log S/CO versus Log IU/mL (Figure 1). Hence, from the Logit-Log regression line, the HBsAg concentrations (IU/mL) in the seroconversion samples were calculated. In Figure 2, we give an example of the course of HBsAg in milli-International Unit (mIU)/mL in the BIO-RAD assay as compared with the HBV-DNA concentration in copies/mL measured with the bDNA 3.0 assay in seroconversion panel #11006. There was a remarkably parallel increase of Log HBsAg concentration and Log HBV-DNA concentration, not only in panel #11006 but also in the other 4 seroconversion panels (#6284, #6289, #6292, and #11008), as illustrated for the two HBsAg assays in Figure 3. Not surprisingly, a strong correlation was found between the HBV-DNA and HBsAg concentrations in the ramp-up phase samples (Figure 4). Combining the data on 25 ramp-up phase samples from the five seroconversion panels together, we calculated a ratio (95% CI) of one HBV virion (or HBV-DNA copy) to 1650 (960–2830) subviral HBsAg particles for the PRISM assay and a particle ratio of 1:1450 (770–2740) for the BIO-RAD assay. The parameters for this calculation are described in the methods and are summarized in Figure 4.

For each of the seroconversion panels the time point where the HBsAg signal crosses the cutoff was calculated from the regression line between Log IU/mL HBsAg and time (Figure 2). The corresponding HBV-DNA concentration at the HBsAg cutoff crossing points was calculated from the HBV-DNA regression line (Figure 2). The geometric mean (and range) of the HBV-DNA concentrations at the HBsAg seroconversion points were 1493 (1057–2169) and 2453 (1456–3521) copies/mL for the PRISM and BIO-RAD HBsAg assays, respectively. Table 1 presents the viral loads and the HBsAg seroconversion time points (or lengths of the infectious pre-HBsAg window period) for the two HBsAg assays for each of the panels.

### 3.2. Comparison of HBV-NAT and HBsAg Conversion Point in Ramp-Up Phase

**Figure 5:**
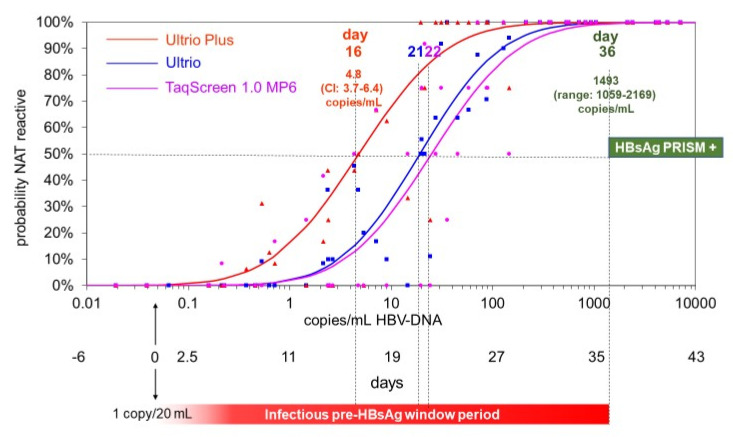
Probability curves calculated from proportions of reactive results obtained by multiple replicate testing (*n* = 4–18, Table 2) of five seroconversion panels with three NAT blood screening methods in a combined probit analysis on all seroconversion panel data. The HBV-DNA concentrations at the time points on the *x*-axis were projected from the measured HBV-DNA concentrations in the bDNA 3.0 assay in each of the five panels separately using the regression analysis parameters presented in Table 1. The given time points of the seroconversion samples were shifted so that the start of the infectious window period (day 0) for each of the five panels was set at a concentration of 1 HBV-DNA copy/20 mL plasma (Table 1). The end of the infectious pre-HBsAg window period (red bar under graph) and the beginning of the HBsAg PRISM reactivity (green bar) were set at the geometric mean HBV-DNA concentration at the HBsAg cutoff crossing points estimated for the five panels individually (Table 1, see example Figure 2).

To compare the HBV-NAT and HBsAg conversion points in the ramp-up phase, we combined the data of the five seroconversion panels for performing probit analyses on the replicate NAT results in the Ultrio, Ultrio Plus, and TaqScreen 1.0 assay (in MP6-dilution), respectively. For each assay the projected HBV-DNA concentration in the ramp-up phase samples was plotted against the proportion of reactive NAT results with 4–18 replicate tests per sample. Figure 5 presents the probit curves of the three NAT methods as compared with the estimated overall PRISM HBsAg conversion time point (based on a mean viral doubling time of 2.44 days), whereby both the time after the start of the infectious window period and the Log HBV-DNA concentration are presented on the *x*-axis. For the probit analysis on the combined dataset of the five seroconversion panels, the estimated 50% NAT detection limits and conversion time points are given in Figure 5 and in Table 1. The pre-HBsAg (PRISM) window period reduction achieved by the Ultrio Plus assay was 20 days as compared with 14–15 days with the Ultrio and TaqScreen 1.0 (the latter in MP6-NAT format).

### 3.3. Comparison of NAT Detection Limits on HBV Genotype A Dilution and Seroconversion Panels

To compare the 50% and 95% LODs of the three NAT systems on the five seroconversion panels (taken together) against the PeliCheck HBV-DNA genotype A reference panel (tested in the same series of experiments), we performed a probit analysis in a parallel line model. This allowed us to compare the 50% and 95% LODs of the NAT methods on the combined seroconversion panel samples versus those on the standard dilution panel (Table 2). The LODs on the dilution and seroconversion panels for each of the NAT methods, as well as the potencies of the two panel types, were comparable (Table 2). This result confirms the equal calibration and correct estimation of HBV-DNA in copies/mL in the samples of the two panel types. Moreover, it confirms the validity of our assumption that the viral doubling time observed by regression analysis on viral loads detected by the bDNA 3.0 assay is the same in the viral load range below the quantification limit of the bDNA 3.0 assay (0.1–1000 copies/mL).

### 3.4. Comparison of NAT Detection Limits on HBV Standard Dilutions of Different Genotype

Table 3 (upper panel) compares the 50% and 95% LODs of the Ultrio Plus and TaqScreen 1.0 assays on the PeliCheck HBV-DNA panels of different genotypes showing opposite variation of LODs in the two assays. In a parallel line model, the TaqScreen 1.0 assay on undiluted samples was between 2.0 (1.0–4.6)- and 3.3 (1.6–8.6)-fold more sensitive than the Ultrio Plus assay on the genotype A, D, and E panels. By contrast, the sensitivity factors of TaqScreen 1.0 relative to Ultrio Plus were opposite on the HBV genotype B, F, and G samples and varied between 0.5 (0.20–1.0) and 0.25 (0.1–0.6). In the lower panel of Table 1, we compared the previous (less sensitive) Ultrio assay with the TaqScreen 1.0 assay in 1:6 dilution (mimicking MP6-NAT)**.** The old Ultrio in individual donation (ID)-NAT format reached significantly higher sensitivity than the TaqScreen 1.0 in MP6-NAT configuration on the genotype B, F, and G samples, but the differences were not significant for the genotype A, C, D, and E panels. The data in Table 3 show that the relative sensitivity factors between two NAT methods on one genotype are not representative of the other genotypes. Because we tested only one standard per genotype it cannot be concluded that it is the genotype that causes the differences (see below).

### 3.5. Analytical Sensitivity of NAT Methods on Multiple Standards per Genotype

Since 2007, BioQControl has manufactured multiple standard dilution panels of different genotypes for NAT performance evaluation studies in several laboratories around the world. We collected the analytical sensitivity data on our HBV genotype standard dilution panels until 2018; Table 4a–d present the probit analysis results grouped per genotype and per standard. For some standards (such as the 2nd WHO 97/750 International Standard), the total number of replicate tests per dilution mounted up to more than 300, while for other standards only 12 replicates were tested.

On HBV genotype A2 standards (Table 4a), the detection limits of the Roche cobas assay versions were consistently and significantly lower than those of the Ultrio Plus and Elite assay versions. The TaqScreen 1.0 assay was also significantly more sensitive than Ultrio Plus on one of the two HBV genotype A1 members of the WHO genotype reference panel but not on the other HBV genotype A1 sample. Pasteurization of the VQC-Sanquin genotype A standard (at a 100-fold dilution in PBS) significantly reduced the analytical sensitivity of the old Ultrio assay, but this difference largely disappeared in the Ultrio Plus and Elite assays due to the target enhancer reagent.

Table 4b compared the LODs of the Ultrio assay versions and TaqScreen 1.0 on different HBV genotype B and C standards. TaqScreen 1.0 tended to be slightly less sensitive on the PeliCheck reference panel but not on the three HBV genotype B members of the WHO reference panel. On four HBV genotype C standards, TaqScreen 1.0 was consistently 2–3-fold more sensitive than Ultrio Plus.

When comparing the LODs on four HBV genotype D and two genotype E standards, TaqScreen 1.0 was consistently 2–3-fold more sensitive than Ultrio Plus (Table 4c). However, TaqScreen 1.0 assay was 2-fold less sensitive than Ultrio Plus on one HBV genotype F standard and the assay seemed to poorly detect the WHO HBV genotype F3 sample (Table 4d). Whether there is indeed a more than 10-fold under-detection by this assay needs to be confirmed in a second experiment. In another rare HBV genotype (genotype G), TaqScreen 1.0 was 4-fold less sensitive, but this was not confirmed on another genotype G sample from the WHO reference panel.

## 4. Discussion

In this study, we carefully quantified HBV-DNA and HBsAg concentrations in ramp-up phase samples of five HBV genotype A seroconversion samples against standards for HBV-DNA genotype A (calibrated in IUs and copies) and for HBsAg adw_2_ (calibrated in IUs and nanograms). Our mathematical analyses of the dynamics of the HBV-DNA and HBsAg concentration in the ramp-up phase of five seroconversion panels clearly demonstrated that both markers for HBV plasma viremia increase with similar doubling times in early infection. This conclusion was based on our observation of a linear correlation between the time in the ramp-up phase and the Log concentration for both markers, as well as on a remarkable parallelism of the two regression lines in each of the seroconversion panels. As a result, we observed a strong correlation between the Log HBV-DNA concentration and Log HBsAg concentration in the ramp-up phase samples with constant ratios of the number of HBV-DNA copies per ng of HBsAg. On the basis of HBsAg standardization work of Prof Gerlich and colleagues [18,19], we were able to estimate that for each potentially infectious HBV virion (or HBV-DNA copy), 1600 (CI: 1000–2800) subviral 20 nm HBsAg particles are produced in the early acute phase of HBV genotype A infection. Similar HBsAg/HBV particle ratios have been found in the acute phase of chimpanzees infected with HBV genotype A and in highly viremic HBV genotype A carriers; however, in Egyptian HBsAg-positive blood donors with borderline detectable HBV-DNA (genotype D), these ratios were found to be a million (thousand to billion) fold higher [12,19].

One could argue that the slope of the Log-linear increase of the HBV-DNA concentration observed in the seroconversion panels above the quantification limit of viral load assay (the bDNA 3.0 assay in our study) may be different in an earlier stage of infection. To investigate this, we tested the earlier ramp-up phase samples below the bDNA 3.0 quantification limit in multiple replicate Ultrio and Ultrio Plus tests. Some of the panels were tested in as many as 16–18 replicates, but in other panels only 4 replicate test results were available. As not enough ramp-up phase samples in the seroconversion panels had viral loads with intermittent reactivity in the Poisson detection endpoint range of the NAT methods (0.1–100 copies/mL), we decided to combine the replicate test results of the five panels. Consequently, there were sufficient data to estimate the 50% and 95% LOD of the Ultrio, Ultrio Plus, and TaqScreen 1.0 assay (tested in MP6 dilution) by probit analysis. The combined probit analysis on all seroconversion samples allowed for comparison of the 50% NAT conversion points with the HBsAg cutoff crossing time points for the PRISM and BIO-RAD assays, whereby a concentration of 1 copy/20 mL plasma in an RBC unit was arbitrarily set as the start of the infectious window period (day 0). This analysis showed that the Ultrio Plus assay in ID-NAT configuration detected early HBV infection 20–22 days before the two HBsAg assays, whereas the previous Ultrio assay and TaqScreen 1.0 assay (the latter in MP-6 NAT format) detected plasma viremia 5–6 days later than the Ultrio Plus assay.

We then compared the probit curves in the combined seroconversion sample analysis with those found on the PeliCheck HBV genotype A standard dilution panel in a parallel line model and found that the LODs on (and the potencies of) the two HBV genotype A panel types were comparable. This confirms that the projected HBV-DNA concentrations in the very early ramp-up phase samples were correctly estimated by the regression analyses in each panel and that the HBV doubling times in the early infected plasma donors were the same in concentrations above and below the quantification limit of the viral load assay. From this analysis, we conclude that the 50% LOD on HBV-DNA genotype A standard dilutions is equivalent to the 50% LOD on HBV genotype A seroconversion panels provided that the quantification in HBV-DNA copies or IUs in both panel types is based on the same standard. In other words, for NAT and antigen assay performance, the standard dilution panels are functionally equivalent to seroconversion panels. Hence, the 50% LODs on standard dilution panels can also be used for modelling the length of the infectious window period with different HBV NAT assays, a concept that was already accepted for the development of window period risk models with different NAT options [17,20]. However, the present study also proves that determination of the cutoff concentration of HBsAg assays on WHO HBsAg genotype A standard dilution series is equivalent to estimation of the HBsAg cutoff crossing point in HBV genotype A seroconversion panels because of the similar exponential (Log-linear) increase of HBsAg and HBV-DNA. Therefore, HBsAg seroconversion panels can also be replaced by standard dilution panels.

The advantage of using standard dilution panels for HBV-DNA and HBsAg is that LODs can be more accurately established on critical concentrations of well-calibrated standards of known stability in contrast with seroconversion panels that often lack enough samples with intermittent NAT reactivity. Moreover, the use of standard dilution panels instead of seroconversion panels allows for comparison of the analytical sensitivity for multiple genotypes, which give variable and opposite results for the NAT assays of two IVD manufacturers as the data in this manuscript show. HBV seroconversion panels are available in limited supply and restricted to the genotypes prevalent in the countries where they are sourced (usually HBV genotype A for the plasma donor seroconversion panels collected in the USA). The analytical sensitivity panel data obtained over a decade and presented in this manuscript demonstrate that the LODs of different HBV-NAT methods on one genotype or on one International Standard are not necessarily representative of other genotypes or even of samples of the same genotype. Similarly, performance evaluation of different HBsAg assays using the WHO HBsAg genotype reference panel calibrated in IUs and nanograms demonstrated that assay detection limits on the 2nd WHO HBsAg genotype A (00/588) standard may not always be predictive for those on the other genotypes [11].

Our quantitative and mathematical analyses revealing similar dynamics of HBV-DNA and HBsAg in early plasma viremia likely hold for viral RNA and antigen in other infections as well. Similar exponential growth curves were also observed for HIV-RNA and p24 antigen and likely also exist for HCV-RNA and core antigen [16,21,22]. In addition, for these markers, we and others demonstrated a strong correlation between viral load and antigen concentration in large clinical sample panels obtained from early infected blood donors in the antibody negative window period [21,22] The validity of the Log-linear ramp-up phase model has been extensively validated for NAT methods [14,15,16] and is the basis for window period risk models [17,20]. The present paper provides evidence that the same models can also be used for estimating the length of the window period and residual risk with viral antigen assays.

Recently, a three-year transition period to full implementation of the IVDR [8] began for which a new version of the Common Specifications (CS) [7] was adopted by the European Commission. This legislation prescribes the performance evaluation criteria for EU market approval of class D IVDs intended for blood screening. We were surprised that the requirements for test performance in this new version of the CS were not innovated compared with the previous version of more than two decades ago. Still, diagnostic sensitivity has to be demonstrated with 10–30 seroconversion panels depending on the analyte, but there are no clear requirements for analytical sensitivity for different genotypes except that the assay performance should be ‘state of the art’. We recommend that IVD manufacturers of new NAT or antigen assays use standard dilution panels of different genotypes rather than seroconversion panels (and only one WHO standard) to demonstrate equivalence or superiority to previous assay versions. Blood safety testing in the EU would be better regulated when the minimum requirement for the analytical sensitivity of NAT blood screening assays is a 95% LOD of 100 copies/mL for the most prevalent genotypes. The analytical sensitivity data in this manuscript show that this sensitivity limit is feasible and required for ‘state of the art’ NAT performance. Similarly, a sensitivity limit expressed in ng/mL (or IU/mL) could be defined in the CS for antigen (or antigen–antibody combination (combo)) assays. Extensive standardization studies of German investigators have demonstrated the suitability of plasma-derived and recombinant HBsAg preparations of different genotypes for performance evaluation of HBsAg assays showing that a detection limit of <0.1 IU/mL or even <0.05 IU/mL can be achieved by ‘state of the art’ automated test systems for blood screening [11,23]. Our study shows that it may even be feasible to use dilution series from early ramp-up phase seroconversion samples as reference panels of different genotypes for defining a viral load in copies/mL that should be detectable by antigen or combo assays. However, for demonstrating the feasibility of this approach, it must be examined first with multiple individual seroconversion samples that the viral load to antigen concentration (copy/ng) ratios are comparable for different genotypes.

In conclusion, our detailed mathematical analyses on HBV dilution and seroconversion panels demonstrate that for NAT and antigen blood screening assays, analytical sensitivity studies with well-calibrated reference panels of different viral genotypes are more precise in predicting the diagnostic sensitivity of IVDs and make obligatory testing of seroconversion panels unnecessary.

## Figures and Tables

**Table 1:** Regression analysis parameters for exponential (Log-linear) increase of HBV-DNA and HBsAg concentration over time in five seroconversion panels based on given sampling times in Zeptometrix data sheet (upper (**a**)) and when time points are adjusted arbitrarily to a start of the infectious window period at 1 HBV-DNA copy per 20 mL plasma/RBC transfusion at day 0 (lower (**b**)).

(a)											
Panel	Reactive Samples in bDNA 3.0 Assay			Calculated Start WP * at 0.05 Copies/mLat Day	Log-Linear Increase HBV-DNA		HBV-DNA Copies/mL at 50% LOD ^			HBV-DNA Copies/mL at HBsAg Cutoff Crossing Point	
	Day Start	Day End	N_meas._		Doubling Time (Days)	Correl. Coeff.	Ultrio Plus	Ultrio	Taq Screen MP6	PRISM	BIO-RAD
6284	50	64	5	17.7	1.81	0.994				1399	1456
6289	31	49	4	−13.2	2.71	0.974				1057	2956
6292	29	52	6	−16.1	2.75	0.987				1782	2132
11006	42	58	6	6.1	2.31	0.995				1298	2750
11008	72	86	4	28.4	2.61	0.995				2169	3521
All panels					2.44 #		4.8	19.2	25.0	1493 $	2453 $
**(b)**											
**Panel**	**Reactive Samples in bDNA 3.0 Assay**			Calculated Start WP * at 0.05 Copies/mL **at Day**	**Log-Linear Increase HBV-DNA**		**HBV-DNA Conversion Time Point (Day)**			**HBsAg Conversion Time Point (Day)**	
	**Day** **Start**	**Day** **End**	**N_meas._**		**Doubling** **Time (Days)**	**Correl.** **coeff.**	**Ultrio Plus**	**Ultrio**	**Taq** **Screen MP6**	**PRISM**	**BIO-RAD**
6284	32.3	46.3	5	0	1.81	0.994				29.8	29.9
6289	44.2	62.2	4	0	2.71	0.974				38.9	43.0
6292	45.1	68.1	6	0	2.75	0.987				42.4	41.7
11006	48.1	64.1	6	0	2.31	0.995				46.1	48.5
11008	43.6	57.7	4	0	2.61	0.995				40.2	42.1
All panels					2.44 #		16.1	20.9	21.9	36.2 $	38.0 $

* WP = window period. ^ 50% LODs (CI) could be calculated only with the five seroconversion panels taken together (Figure 5) and were 4.8 (3.7–6.4), 19.2 (15.6–23.7), and 25 (17–36) Copies/mL. # mean value. $ geometric mean value.

**Table 2:** Comparison of NAT conversion points with three test systems on five seroconversion (SC) panels with LODs on HBV genotype A standard dilution panel using parallel line probit analysis.

NAT System	HBV Panels #	Samples	Replicates	50% LOD (Copies/mL)	95% LOD (Copies/mL)	Potency (CI) Dilution to SC Panels
Ultrio	5 SC	69	5–18	19.5 (14.8–25.5)	208(140–347)	1.04 (0.62–1.74)
	dilution	10	24	18.7 (12.1–28.9)	200 (120–375)	
Ultrio Plus	5 SC	69	4–16	4.6 (3.4–6.3)	60.5 (38.4–109.7)	0.81 (0.41–1.56)
	dilution	10	12	5.7 (3.2–10.25)	74.8 (38.8–166.4)	
s201 1:6 ^	5 SC	69	4	31.3 (20.0–49.2)	380 (206.3–913)	1.84 (0.63–8.85)
	dilution	10	12	17.0 (9.5–30.22)	207 (104–527)	

# HBV seroconversion (SC) and standard dilution panels were tested in multiple replicates in Ultrio and TaqScreen 1.0 in 1:6 dilution in a head-to-head comparison study in France [2]. The same panels were also later tested in multiple replicates using the Ultrio and Plus assays (data kindly provided by Dr. J. Linnen, Gen-Probe, currently Grifols). ^ TaqScreen 1.0 on s201 platform was performed on 1:6 dilutions of panel members to mimic MP6-NAT.

**Table 3:** Analytical sensitivity of TaqScreen 1.0 relative to Ultrio Plus (upper table) and previous Ultrio version relative to TaqScreen 1.0 in MP6 format (lower table).

Genotype	50% LOD ^ Ultrio Plus (*n* = 12)	50% LOD ^ TaqScreen 1.0 (*n* = 12)	95% LOD ^ Ultrio Plus (*n* = 12)	95% LOD ^ TaqScreen 1.0 (*n* = 12)	Relative Sensitivity Taqscreen to Ultrio Plus #
A	5.7 (3.5–9.4)	2.9 (1.7–4.8)	41.4 (24.3–77.2)	27.1 (15.2–52.7)	2.01 (0.96–4.62)
B	3.1 (1.9–5.2)	6.4 (3.7–10.9)	22.7 (13.3–42.4)	60.2 (33.5–119)	0.49 (0.21–1.05)
C	4.2 (2.6–7.0)	3.0 (1.8–5.2)	30.6 (17.7–57.8)	28.6 (15.9–56.9)	1.43 (0.63–3.60)
D	5.2 (3.1–8.6)	1.6 (0.9–2.8)	37.4 (18.3–70.5)	14.7 (7.9–29.6)	3.28 (1.56–8.61)
E	4.7 (2.8–7.8)	2.1 (1.2–3.8)	33.8 (19.6–63.7)	20.1 (10.9–40.2)	2.28 (0.97–5.90)
F	2.6 (1.5–4.3)	5.4 (3.1–9.2)	18.5 (10.8–34.8)	50.7 (28.1–100)	0.48 (0.21–0.97)
G	1.9 (1.1–3.1)	7.2 (4.2–12.3)	13.5 (7.8–25.3)	68.1 (38.0–134)	0.25 (0.09–0.59)
**Genotype**	**50% LOD ^** **Ultrio** **(*n* = 24)**	**50% LOD ^** **TaqScreen 1.0 MP6** **(*n* = 12)**	**95% LOD ^** **Ultrio** **(*n* = 24)**	**95% LOD ^** **TaqScreen 1.0 MP6** **(*n* = 12)**	**Relative Sensitivity Ultrio to Taqscreen MP6 #**
A	18.6 (13.0–26.8)	17.2 (10.1–29.1)	147 (99–228)	162.3 (91.3–316)	0.92 (0.31–2.08)
B	5.4 (3.7–7.7)	38.1 (22.4–65.2)	42.2 (28.5–64.9)	361 (201–716)	7.14 (3.73–13.95)
C	10.0 (6.9–14.4)	18.1 (10.6–31.2)	78.5 (52.5–122)	172 (95–341)	1.78 (0.57–4.05)
D	15.4 (10.6–22.2)	9.3 (5.1–16.7)	121 (81.0–188)	87.9 (47.4–177.6)	0.62 (0.16–1.69)
E	9.3 (6.6–13.1)	12.7 (7.0–22.8)	73.1 (49.7–112)	121 (65.6–241)	1.30 (0.48–2.91)
F	11.9 (8.3–17.2)	32.1 (18.6–55.5)	93.8 (63.0–146)	304 (168–602)	2.69 (1.25–5.04)
G	4.6 (3.2–6.6)	43.2 (25.2–73.8)	36.2 (24.5–55.7)	409 (228–805)	9.25 (4.88–19.35)

^ LODs calculated by parallel line probit analysis comparing different HBV genotypes in one assay. # relative sensitivities calculated by parallel line probit analysis comparing two assays per single genotype.

**Table 4:** Detection limits of different Procleix Ultrio and cobas MPX versions calculated by probit analysis on data reported by laboratories that tested the analytical sensitivity panels of different genotypes manufactured from calibrated standards in Copies/mL by BioQControl. (**a**) Detection limits on HBV-DNA genotype A standard dilution panels (**b**) Detection limits on HBV-DNA genotype B and C standard dilution panels. (**c**) Detection limits on HBV-DNA genotype D and E standard dilution panels. (**d**) Detection limits on HBV-DNA genotype F and G standards.

(a)					
HBV-DNA Standard	Panel	Assay	*n*	50% LOD (CI) Copies/mL	95% LOD (CI) Copies/mL
S0010 Eurohep HBV-DNA genotype A2	P0001	Ultrio	48	9.4 (5.0–18.0)	93.9 (40.9–493)
	P0001	Ultrio Plus	96	3.6 (2.9–4.4)	40.4 (29.2–60.2)
	P0001	Ultrio Elite	24	7.9 (5.5–11.2)	49.1 (29.4–116)
	P0001	TaqScreen 1.0	12	2.3 (1.3–3.8)	14.1 (7.2–56.6)
	P0272	cobas MPX	48	1.7 (1.0–2.4)	10.3 (6.2–28.8)
WHO HBV-DNA 97/750 genotype A2 #	P0023	Ultrio	32	13.1 (6.3–32.0)	101 (38.7–1020)
	P0023	Ultrio Plus	303	4.4 (3.3–5.9)	28.4 (18.0–57.7)
	P0023	Ultrio Elite	252	4.4 (3.6–5.4)	30.9 (22.4–47.4)
	P0023	cobas MPX	12	1.8 (0.93–2.8)	8.0 (4.4–37.4)
S0011 VQC-Sanquin HBV-DNA genotype A2	S2384	Ultrio	48	27.2 (14.3–105)	235 (153–407)
	P0007	Ultrio	24	15.7 (7.0–33.9)	208 (77.6–2022)
	S2384	Ultrio Plus	12	5.7 (3.4–9.7)	49.4 (27.4–103)
	P0007	Ultrio Plus	48	4.8 (3.7–6.2)	38.8 (25.6–68.5)
	P0007	Ultrio Elite	74	3.4 (2.3–4.8)	43.2 (24.8–98.0)
	S2384	TaqScreen 1.0	12	2.8 (1.7–4.8)	24.5 (13.7–50.9)
	P0007	cobas MPX	24	1.9 (1.3–2.7)	13.0 (7.7–29.6)
S0043 VQC-Sanquin HBV-DNA genotype A2 heat-inactivated	P0031	Ultrio	58	56.5 (31.5–104)	715 (316–3046)
	P0031	Ultrio Plus	24	6.6 (2.7–17.4)	64.2 (22.4–109)
	P0031	Ultrio Elite	25	5.7 (4.0–8.2)	40.8 (24.3–91.7)
	P0031	cobas MPX	12	2.4 (1.4–4.2)	18.6 (9.1–75.9)
	P0251	TaqScreen 2.0	12	2.8 (1.5–4.3)	23.8 (12.4–99.3)
WHO HBV-DNA genotype A2 5086/08-3	P0108	Ultrio Plus	12	3.0 (1.8–5.1)	22.1 (12.7–40.8)
	P0108	TaqScreen 1.0	12	1.7 (1.1–2.8)	9.5 (5.7–16.8)
WHO HBV-DNA genotype A1 5086/08-1	P0106	Ultrio Plus	12	6.3 (3.7–10.7)	22.9 (13.2–41.9)
	P0106	TaqScreen 1.0	12	1.8 (1.1–2.9)	9.8 (6.0–17.0)
WHO HBV-DNA genotype A1 5086/08-2	P0107	Ultrio Plus	12	4.3 (2.5–7.3)	31.4 (17.9–58.5)
	P0107	TaqScreen 1.0	12	6.3 (3.7–10.7)	34.4 (19.8–63.7)
**(b)**					
**HBV-DNA Standard**	**Panel**	**NAT Method**	***n***	**50% LOD (CI) Copies/mL**	**95% LOD (CI) Copies/mL**
S0098 BioQ HBV-DNA genotype B	S2385	Ultrio	24	5.3 (3.6–7.8)	49.9 (30.7–94.2)
	P0009	Ultrio	12	3.3 (1.9–5.5)	23.6 (13.6–45.9)
	S2385	Ultrio Plus	12	3.1 (1.8–5.4)	29.4 (16.0–62.8)
	P0009	Ultrio Plus	36	2.8 (2.1–3.8)	20.3 (13.6–45.9)
	P0009	Ultrio Elite	18	2.7 (1.8–4.1)	34.5 (22.1–58.0)
	S2385	TaqScreen 1.0	12	6.4 (3.7–10.9)	59.5 (32.1–129)
WHO HBV-DNA genotype B1 5086/08-4	P0109	Ultrio Plus	12	5.0 (3.1–8.2)	36.6 (21.9–64.7)
	P0109	TaqScreen 1.0	12	3.8 (2.4–6.1)	20.9 912.8–35.9)
WHO HBV-DNA genotype B2 5086/08-5	P0110	Ultrio Plus	12	3.6 (2.2–6.1)	26.7 (15.6–48.4)
	P0110	TaqScreen 1.0	12	3.5 (2.2–5.6)	19.3 (11.9–33.0)
WHO HBV-DNA genotype B4 5086/08-6	P0111	Ultrio Plus	12	4.4 (2.6–7.2)	32.1 (19.0–57.4)
	P0111	TaqScreen 1.0	12	3.3 (2.1–5.2)	18.0 (11.1–30.8)
S0057 BioQ HBV-DNA genotype C	S2386	Ultrio	24	10.0 (6.4–15.5)	64.3 (37.8–137)
	P0010	Ultrio	12	14.3 (6.3–32.9)	115 (48.7–368)
	S2386	Ultrio Plus	12	4.2 (2.3–7.8)	27.3 (14.0–66.9)
	P0010	Ultrio Plus	36	4.6 (2.9–7.6)	37.4 (20.3–93.4)
	P0010	Ultrio Elite	18	5.0 (2.4–10.2)	40.0 (18.6–114)
	S2386	TaqScreen 1.0	12	2.9 (1.6–5.4)	19.0 (9.7–47.2)
WHO HBV-DNA genotype C2 5086/08-7	P0112	Ultrio Plus	12	7.3 (4.3–12.3)	53.2 (30.6–97.9)
	P0112	TaqScreen 1.0	12	2.4 (1.5–3.8)	13.2 (8.1–22.6)
WHO HBV-DNA genotype C2 5086/08-8	P0113	Ultrio Plus	12	4.0 (2.3–6.7)	29.2 (16.9–53.2)
	P0113	TaqScreen 1.0	12	2.4 (1.5–3.9)	13.2 (7.9–23.1)
WHO HBV-DNA genotype C2 5086/08-9	P0114	Ultrio Plus	12	3.5 (2.1–5.9)	25.6 (14.9–46.6)
	P0114	TaqScreen 1.0	12	1.6 (1.0–2.6)	8.7 (5.1–15.4)
**(c)**					
**HBV-DNA Standard**	**Panel**	**NAT Method**	***n***	**50% LOD (CI) Copies/mL**	**95% LOD (CI) Copies/mL**
S0107 Eurohep HBV-DNA genotype D	P0002	Ultrio	48	3.5 (2.2–5.8)	20.9 (11.0–72.0)
	P0002	Ultrio Plus	48	2.2 (1.1–4.0)	25.3 (10.9–142)
S0058 BioQ HBV-DNA genotype D	S2387	Ultrio	24	15.2 (11.0–21.2)	80.8 (53.1–144)
	P0011	Ultrio	12	14.9 (6.7–32.9)	123 (53.3–371)
	S2387	Ultrio Plus	12	5.1 (3.2–8.3)	27.5 (16.4–53.7)
	P0011	Ultrio Plus	36	4.6 (2.9–7.3)	37.9 (20.9–91.1)
	P0011	Ultrio Elite	18	5.3 (2.8–10.2)	44.1 (21.6–118)
	S2387	TaqScreen 1.0	12	1.6 (1.0–2.6)	8.4 (4.9–16.6)
WHO HBV-DNA genotype D1 5086/08-10	P0115	Ultrio Plus	12	4.8 (2.8–7.9)	34.9 (20.4–63.0)
	P0115	TaqScreen 1.0	12	1.6 (0.9–2.7)	8.7 (5.2–15.4)
WHO HBV-DNA genotype D3 5086/08-11	P0116	Ultrio Plus	12	3.5 (2.1–6.0)	25.9 (14.9–47.9)
	P0116	TaqScreen 1.0	12	1.0 (0.6–1.6)	5.3 (3.2–9.2)
WHO HBV-DNA genotype D1 5086/08-12	P0117	Ultrio Plus	12	2.7 (1.6–4.5)	19.6 (11.4–35.4)
	P0117	TaqScreen 1.0	12	1.5 (0.9–2.5)	8.1 (4.8–14.3)
S0059 BioQ HBV-DNA genotype E	S2388	Ultrio	24	9.4 (6.3–14.0)	110 (64.3–225)
	P0012	Ultrio	12	11.4 (7.3–17.9)	58.6 (35.9–107)
	S2388	Ultrio Plus	12	4.6 (2.6–8.3)	54.2 (28.0–124)
	P0012	Ultrio Plus	36	3.2 (2.4–4.1)	16.2 (11.3–26.1)
	P0012	Ultrio Elite	18	2.5 (1.7–3.7)	12.7 (8.2–21.7)
	S2388	TaqScreen 1.0	12	2.1 (1.1–3.8)	23.9 (12.2–54.3)
WHO HBV-DNA genotype E1 5086/08-13	P0118	Ultrio Plus	12	4.3 (2.6–7.2)	31.9 (18.7–57.4)
	P0118	TaqScreen 1.0	12	1.6 (0.9–2.9)	8.7 (5.2–15.4)
**(d)**					
**HBV-DNA Standard**	**Panel**	**NAT Method**	***n***	**50% LOD (CI) Copies/mL**	**95% LOD (CI) Copies/mL**
S0060 BioQ HBV-DNA genotype F	S2389	Ultrio	24	11.9 (8.4–16.9)	78.8 (50.5–143)
	P0013	Ultrio	12	11.2 (6.8–18.6)	82.2 (47.2–161)
	S2389	Ultrio Plus	12	2.6 (1.6–4.2)	17.0 (9.8–34.4)
	P0013	Ultrio Plus	36	2.7 (2.0–3.6)	19.8 (13.3–33.1)
	P0013	Ultrio Elite	18	3.8 (2.5–5.7)	27.6 (17.0–50.1)
	S2389	TaqScreen 1.0	12	5.3 (3.3–8.8)	35.2 (20.2–71.6)
WHO HBV-DNA genotype F3 5086/08-14	P0119	Ultrio Plus	12	1.5 (1.0–2.6)	11.0 (6.2–20.3)
	P0119	TaqScreen 1.0	12	22.2 (13.4–37.0) ^	122 (71.4–222) ^
S0061 BioQ HBV-DNA genotype G	S2390	Ultrio	24	4.6 (3.1–6.7)	44.3 (29.9–86.1)
	P0014	Ultrio	12	6.2 (3.8–10.0)	36.0 (21.3–67.9)
	S2390	Ultrio Plus	12	1.8 (1.0–3.2)	17.7 (9.5–38.7)
	P0014	Ultrio Plus	36	2.6 (2.0–3.5)	15.3 (10.6–25.0)
	P0014	Ultrio Elite	18	2.9 (2.0–4.3)	17.0 (10.9–29.7)
	S2390	TaqScreen 1.0	12	7.2 (4.2–12.4)	96.6 (37.3–154)
WHO HBV-DNA genotype G 5086/08-15	P0120	Ultrio Plus	12	4.8 (2.8–8.2)	35.2 (20.2–64.9)
	P0120	TaqScreen 1.0	12	3.3 (2.0–5.4)	18.1 (10.9–31.6)

# 1 IU = 5.33 copies. ^ data to be confirmed in another experiment.

## Data Availability

Data are collected by BioQControl and are available on request.
